# Independent predictors of clinical outcomes after surgical correction of degenerative lumbar scoliosis: a retrospective cohort study

**DOI:** 10.3389/fsurg.2026.1820539

**Published:** 2026-08-25

**Authors:** Xiao Xiong, Feng Wang, Ruiling Wang, Tong Tong

**Affiliations:** Department of Spine Surgery, The Third Hospital of Hebei Medical University, Shijiazhuang, China

**Keywords:** clinical prognosis, degenerative lumbar scoliosis, posterior decompression and fusion, predictive parameters, risk factors

## Abstract

**Objective:**

To identify the independent risk factors and predictive parameters for clinical outcomes in patients undergoing surgical intervention for degenerative lumbar scoliosis (DLS).

**Methods:**

A retrospective analysis was conducted on 134 patients with DLS who underwent posterior decompression and fusion. Clinical prognosis was evaluated using the Oswestry Disability Index (ODI). Patients were categorized into “better prognosis” and “poor prognosis” groups based on postoperative functional recovery. Potential risk factors—including patient demographics, surgical data, and radiographic/imaging parameters—were analyzed using multivariate analysis. Receiver operating characteristic (ROC) curves were utilized to determine optimal threshold values for predicting surgical efficacy.

**Results:**

One hundred and thirty-four patients with degenerative lumbar scoliosis who underwent posterior surgery were included in this study. The analysis showed that the subcutaneous fat tissue thickness (SFTT)**,** MRI-based vertebral bone quality (VBQ), Quantitative Computed Tomography (QCT)**,** degree of kyphosis correction, sagittal balance correction distance were independent predictors of poor prognosis. ROC curve analysis showed that correction degree of kyphosis >20.23°, sagittal balance correction distance >2.92 cm, QCT >104.6 mg/cm^3^, VBQ <2.88, SFTT <13.5 mm were indicating good clinical efficacy.

**Conclusions:**

Posterior decompression and fixed fusion significantly alleviate pain and enhance quality of life in patients with DLS. Adequate correction of kyphosis and sagittal balance is paramount for achieving optimal functional outcomes. Furthermore, our findings that SFTT, VBQ, and QCT are significantly associated with prognosis suggest that aggressive anti-osteoporosis treatment and early paraspinal muscle exercises may be beneficial for improving long-term surgical success. Future interventional studies are warranted to confirm this potential causal relationship.

## 1 Introduction

Degenerative scoliosis is a three-dimensional deformity of the spine caused by degenerative changes in the lumbar intervertebral disc and articular processes (1, 2). Unlike idiopathic scoliosis, patients with this condition typically experience persistent low back and leg pain, as well as intermittent claudication, prompting them to seek medical attention (3–5). With an increasing emphasis on health-related quality of life, surgical intervention is being pursued by a growing number of patients. The surgical approach effectively relieves compression of the nervous tissue, provides stability to the decompressed segment, prevents instability and corrects scoliosis (6).

Large scale of prospective studies have reported significant improvement in quality of life after surgery for degenerative scoliosis over a follow-up period of at least 2 years (7). However, complex types of kyphosis are accompanied by degeneration and underlying diseases in advanced age, leading to a significant increase in postoperative complications and unsatisfactory postoperative outcomes (7). There are numerous factors that may affect patients with degenerative scoliosis. The implementation of posterior corrective surgery has shown positive outcomes for patients with degenerative scoliosis, achieving good correction in both coronal and sagittal balance (8). Studies have indicated that improvement in coronal imbalance alone does not significantly impact outcome scores, emphasizing the importance of sagittal balance on overall health status (8). However, there is still a lack of research on the prognosis of degenerative scoliosis with a large sample size and comprehensive imaging parameters.

Apart from imaging parameters, the initial condition of the patient also plays a crucial role in determining clinical prognosis. Previous research (9) has demonstrated that bone density affects fusion rates in posterior lumbar fusion surgery, ultimately influencing the patient's prognosis. In recent years, the application of CT scanning raw data for Quantitative Computed Tomography (QCT) analysis has been proposed, which provides a more accurate evaluation of bone without additional examinations (10). More recently, MRI-based vertebral bone quality (VBQ) has shown advantages in assessing bone density (11, 12). By analyzing MRI data, VBQ can accurately evaluate the quality of spinal vertebrae by identifying decreases in bone density and increases in bone trabeculae with fat infiltration (13), and recently studies have suggested that increased VBQ may lead to higher subsidence rates in spinal fusion surgery which may affect the outcome after surgery (11). Lumbar paraspinal muscles play a significant role in lower back pain following spinal surgery (14). Patients with higher lumbar muscularity have shown greater improvement in the Oswestry Disability Index (ODI) after surgery compared to those with lower muscularity (15). Recent studies have shown that subcutaneous fat tissue thickness (SFTT) at the lumbar spine level is more effective than BMI in predicting severe changes in lower back pain, which may have some guiding significance for the prognosis of lumbar spine surgery (16). Moreover, the type of occupation also affects the time required for patients to return to work after transforaminal lumbar interbody fusion surgery, with physically demanding jobs requiring a longer recovery period (17).

The posterior lumbar decompression and internal fixation surgery has shown numerous advantages in treating degenerative scoliosis, the daily life treatment of patients has been greatly improved. However, some patients still experience postoperative stubborn low back pain and lower limb radiation pain, resulting in unsatisfactory postoperative outcomes. This study aims to provide a comprehensive review of patients with degenerative scoliosis who underwent long-stage fixed fusion and analyze the risk factors associated with prognosis. The findings aim to serve as a theoretical basis for improving prognostic outcomes in such patients. The analysis focused on identifying significant factors that affect outcomes which measured by the ODI score within these patient groups.

## 2 Materials and methods

### 2.1 Ethics statement

The retrospective study obtained approval from the Ethics Committee of the Third Hospital of Hebei Medical University in China.

### 2.2 Study design and patient population

This retrospective study included data from patients with degenerative scoliosis who underwent surgery at the Department of Spine Surgery, Third Hospital of Hebei Medical University, Hebei, China, between January 2020 to June 2022. Inclusion criteria were as follows: (1) age between 50 and 80 years; (2) clear diagnosis of degenerative scoliosis based on history, clinical symptoms, and imaging, with the main bending angle between 30 and 60 degrees; (3) all patients underwent posterior approach surgery with posterior pedicle screws combined with 1–3 level interbody fusion; (4) patients received adequate conservative treatment before surgical intervention. All surgeries are performed by two same experienced surgeons.

Exclusion criteria were as follows: (1) history of other types of scoliosis (metabolic, inflammatory, trauma-induced, congenital, or idiopathic); (2) history of lumbar spine surgery; (3) presence of other comorbidities such as neoplasia, trauma, infection, severe osteoporosis; (4) patients lost to follow-up; (5) secondary surgeries due to various reasons, including failed internal fixation, internal fixation joint area slip, and severe intervertebral disc degeneration.

### 2.3 Parameter measurement

According to the measurement methods in previous literature (18, 19), all patients underwent measurements of relevant parameters before and after surgery. All data was measured by an orthopedic surgeons and radiologist. A Kappa value was calculated for assessing the reliability of diagnosis as follows:0–0.2 representing slight agreement, 0.21–0.4 fair agreement, 0.41–0.6 moderate agreement, 0.61–0.8 substantialagreement, and 0.81–1 excellent agreement.

### 2.4 Oswestry disability Index score

The ODI score is used to assess low back pain and functional impairment (7). It consists of ten questions covering aspects that affect daily life. Participants select the description that best reflects their situation for each question and assign a score from 0 to 5. The final ODI score is calculated by summing the scores for all questions, dividing by 50, and multiplying by 100. The ODI score ranges from 0 to 100, with higher scores indicating more severe functional impairment. Postoperative improvement is estimated based on the improvement rate (RR), calculated as (preoperative ODI score—postoperative ODI score) divided by preoperative ODI score, multiplied by 100%. Excellent recovery is defined as a score of 75% to 100%, good recovery as 50% to 74%, fair recovery as 25% to 49%, and poor recovery as 0% to 24%. In this study, a poor surgical outcome was defined as a recovery rate of less than 25%, the remaining patients (recovery rate greater than or equal to 25%) are defined as the good group. All studies were independently rated by two reviewers with complete agreement between raters.

### 2.5 Relevant factors

Data collected included: (1) general patient status: gender, age, BMI, disease duration, occupation, smoking history, comorbidities (e.g., hypertension, diabetes, cardiovascular disease, liver and kidney dysfunction), bone mineral density（DXA method for all vertebrae with fusion segments）, Referring to previous research (20), the VBQ score was calculated with the median of signal intensities in fusion segments vertebral bodies divided by the signal intensity of cerebrospinal fluid at L3. To ensure data homogeneity and eliminate inter-scanner variability, all patients underwent lumbar spine MRI on the same 1.5 T scanner (Magnetom Aera, Siemens Healthineers) using a standardized sagittal T1-weighted sequence. Imaging parameters—including repetition time 455 ms, echo time 8.40 ms, slice thickness 4.0 mm, and a fixed field of view—were kept consistent across all subjects. This protocol effectively minimized baseline signal fluctuations and geometric distortions, ensuring the stability and reproducibility of subsequent VBQ measurements. Paravertebral muscle status range from 0 to 4 scores (14) (mean value of fat to muscle area ratio of the multifidus, erector spinae, and psoas major muscles at each segment of surgery). QCT uses previous methods for detection (10), CT data was analyzed using MW QCT version 6 BMD measurement and analysis system software (Mindways Software Inc, Austin, TX, USA). Select the middle layer of the lumbar spine image axis in the coronal and sagittal planes, delineate the area of interest in the center of the axis, avoid the bone island, bone cortex, and posterior central vein of the vertebral body, and include as much cancellous bone as possible. And calculate the average value of multiple vertebral bodies to obtain the results. Subcutaneous fat tissue thickness (SFTT) was measured as the vertical distance from the tip of spinous process to the skin on axial T2-weighted lumbar spine MRI. Preoperative occupations and work status were established at interviews, referring to previous methods, patients are divided into heavy labor and light labor based on different work intensities (17). (2) surgery-related data: surgery time, bleeding volume, number of fixed fusion segments; (3) imaging parameters: preoperative scoliosis, scoliosis correction, preoperative kyphosis angle, kyphosis correction angle, preoperative coronal balance distance, coronal balance correction distance, preoperative sagittal balance distance, sagittal balance correction distance, preoperative vertebral rotation grading (21), degree of postoperative rotation correction (21), loss of scoliosis correction (end follow-up), the number of degrees of loss of correction of kyphosis (last follow-up), and the fusion rate of L5-S1.

### 2.6 Statistical analysis

Descriptive analyses were performed using means, standard deviations (SD), frequencies for continuous variables, and percentages for categorical and discrete variables. Chi-square or Fisher's exact tests were used for categorical or discrete variables, and independent sample t-tests were used for continuous variables. Multivariate logistic regression was conducted to assess the independent relationship of each explanatory variable. Factors with a *p*-value < 0.05 from the univariate analysis were included in the multivariate logistic model. Receiver operating characteristic (ROC) curves were constructed to evaluate the sensitivity and specificity of risk factors in predicting unfavorable postoperative surgical outcomes. Statistical significance was defined as *p* < 0.05. MedCalc software 22.019 (MedCalc Software Ltd, Ostend, Belgium) was used for all analyses. To rigorously evaluate the predictive performance and overfitting risk of the multivariable logistic regression model, this study employed two internal validation methods. A 10-fold cross-validation was performed by iteratively training the model on nine subsets and validating on the remaining subset, with the mean AUC from the 10 runs representing generalization performance. Additionally, bootstrap validation with 1,000 resamples was conducted to calculate the optimism (AUC difference between bootstrap and original samples) and derive a bias-corrected AUC. All analyses were performed using R software (version 4.5.2, R Foundation for Statistical Computing, Vienna, Austria) utilizing the “caret” package for cross-validation and custom scripts for bootstrap validation.

## 3 Results

### 3.1 General data of patients and comparison of clinical and radiological data

After initial selection and screening of patients, finally One hundred and thirty-four patients with degenerative lumbar scoliosis were included in this study. There were 36 male and 98 female with a mean age of 59.4 ± 7.8 years. The average duration of symptoms was 21.3 months, ranging from 0.7 months to 3.5 years. The mean duration of follow-up was 2.3 years, ranging from 1.5 to 3.2 years. The general information of the collected patients is as follows (Table 1). According to the ODI score at the last follow-up, among all participating patients ODI improved by an average of 18 points (41.7%). The improvement rate was above 25% (Good group) in 103 cases and below 25% (Poor group) in 31 cases.

**Table 1:** General information of patients.

Variable	Value
Mean age(years)	59.4 ± 7.8
Sex	36 male and 98 female
BMI(kg/m^2^)	26.3 ± 4.7
duration of symptoms(months)	21.3 ± 15.2
duration of follow-up(years)	2.3 ± 0.85
Fixed segments(n)	7.4 ± 3.7
Fusion segments(n)	2.7 ± 1.7
Average scoliosis angle(°)	37.8 ± 8.9°

Values are presented as mean ± standard deviation. Statistical signifcance was set at *P* < 0.05.

Two different groups showed that QCT, VBQ, SFTT, correction of kyphosis, sagittal balance correction distance showed statistical differences (*P* < 0.05). In contrast, there were no significant differences between the two groups in terms of parameters related to gender, BMI, disease duration, occupation, comorbidities, operative time, bleeding, correction degree of scoliosis, coronal balance correction distance, number of fixed fused segments, lateral convexity and vertebral rotation(*P* > 0.05) (Table 2).

**Table 2:** Comparison of patient characteristics between good and poor outcome groups.

Variable	Good(*n* = 103)	Poor(*n* = 31)	*P*-value
Age(y)	57.5 ± 8.2	62.3 ± 7.9	0.436
Male sex (female, %)	79 (76.7%)	19 (61.3%)	0.132
BMI(kg/m^2^)	26.7 ± 5.9	25.3 ± 5.6	0.426
Duration of symptoms (m)			0.333
<1	54	16	
1–3	37	9	
>3	12	6	
Occupation			0.337
Light labor(n, %)	45 (44%)	10 (32%)	
Heavy labor(n, %)	58 (56%)	21 (68%)	
Smoking(n, %)	37 (36%)	9 (29%)	0.139
Hypertension (n, %)	29 (28%)	16 (26%)	0.838
Diabetes (n, %)	21 (20%)	13 (21%)	0.888
Cardiovascular disease (n, %)	24 (23%)	12 (25%)	0.851
Liver or kidney dysfunction (n, %)	7 (7%)	2 (5%)	0.339
Bone mineral density(T score)	−0.92 ± 1.42	−1.13 ± 1.25	0.226
QCT(mg/cm^3^)	114.7 ± 11.7	87.3 ± 10.4	**0** **.** **001**
VBQ	3.14 ± 0.63	3.47 ± 0.49	**0** **.** **011**
Muscle fat infiltration classification	2.9 ± 0.4	3.3 ± 0.51	0.464
SFTT(mm)	8.1 ± 2.7 mm	14.2 ± 5.4 mm	**0** **.** **001**
Modic change or not			0.581
0	62	18	
1–2 segments	34	9	
≥3 segments	7	4	
Operative time(min)	316.5 ± 106.6	293.2 ± 117.3	0.436
Blood loss(mL)	904.1 ± 325.4	849.4 ± 359.2	0.242
Fixed segments	7.5 ± 3.7	7.9 ± 3.6	0.361
fusion segments	2.8 ± 1.6	2.6 ± 1.7	0.575
Preoperative scoliosis angle(°)	36.7 ± 9.2	39.3 ± 8.7	0.797
Correction degree of scoliosis(°)	22.7 ± 4.0	20.6 ± 5.3	0.620
Preoperative kyphosis angle(°)	37.3 ± 5.6	35.0 ± 4.7	0.882
Correction degree of kyphosis (°)	30.1 ± 7.6	16.4 ± 8.5	**0** **.** **001**
Preoperative Coronal balance distance(cm)	3.1 ± 1.7	3.6 ± 1.5	0.259
Coronal balance correction distance(cm)	1.4 ± 0.6	1.5 ± 0.4	0.366
Preoperative Sagittal balance distance(cm)	6.4 ± 2.6	5.9 ± 3.5	0.086
Sagittal balance correction distance(cm)	5.9 ± 2.2	2.1 ± 1.5	**0** **.** **001**
Preoperative Nash/Moe index of vertebrae	3.2 ± 1.4	2.8 ± 1.5	0.168
Nash/Moe index of vertebrae correction	1.4 ± 1.3	1.6 ± 1.4	0.100
Loss of degrees for posterior convexity correction(°)	1.7 ± 0.9	2.0 ± 0.4	0.583
Loss of degrees of scoliosis correction(°)	1.1 ± 0.6	1.9 ± 0.7	0.431
L5-S1 fused (n, %)	47 (45.6%)	19(61.3%)	0.126

QCT, quantitative computed tomography; VBQ, MRI-based vertebral bone quality; SFTT, subcutaneous fat tissue thickness; Values are presented as mean ± standard deviation or number (%). Statistical signifcance was set at *P* < 0.05. Bold values indicate statistically significant results (*P*< 0.05).

### 3.2 Reliability of diagnosis

Assessment on parameters was done by an orthopedic surgeons and radiologist. The kappa values obtained from the measurements of all parameters are all significantly greater than 0.9, showing excellent agreements.

### 3.3 Factor related logistic regression analysis

All possible prognostic risk factors were first analyzed through univariate logistic regression analysis. In univariate logistic regression analysis, the QCT, VBQ, SFTT, degree of correction of kyphosis, sagittal balance correction distance were related predictors of prognosis which show statistical differences (*P* < 0.05). However, after univariate logistic regression, there was no statistically significant difference in correction degree of scoliosis and the coronal balance correction distance (*P* > 0.05). Part of the data was presented in Table 3.

**Table 3:** Related factors for good outcome: simple factor logistic regression analysis.

Variable	OR (95% CI)	*P*-value
QCT(mg/cm^3^)	1.204 (1.126–1.288)	**0**.**001**
VBQ	0.404 (0.202–0.808)	**0**.**002**
SFTT(mm)	0.690 (0.602–0.791)	**0**.**001**
Correction degree of kyphosis(°)	1.223 (1.136–1.316)	**0**.**001**
Sagittal balance correction distance(cm)	2.058 (1.511–2.803)	**0**.**001**
Correction degree of scoliosis(°)	1.458 (0.474–1.992)	0.645
Coronal balance correction distance(cm)	1.173 (0.654–1.703)	0.428

QCT, quantitative computed tomography; VBQ, MRI-based vertebral bone quality; SFTT, subcutaneous fat tissue thickness; OR, odds ratio; CI, confidence interval. Statistical signifcance was set at *P* < 0.05. Bold values indicate statistically significant results (*P*< 0.05).

In multivariate logistic regression analysis (show in Table 4), the degree of correction of kyphosis (OR = 1.399, *P* = 0.021), sagittal balance correction distance (OR = 2.634, *P* = 0.011), QCT (OR = 1.401, *P* = 0.008), VBQ (OR = 0.076, *P* = 0.037), SFTT (OR = 0.410, *P* = 0.043) were independent predictors of prognosis (*P* < 0.05).

**Table 4:** Related factors for good outcome: multivariate logistic regression analysis.

Variable	OR (95% CI)	*P*-value
QCT(mg/cm^3^)	1.401 (1.091 −1.799)	0.008
VBQ	0.076 (0.006–0.864)	0.037
SFTT(mm)	0.410 (0.173–0.973)	0.043
Correction degree of kyphosis (°)	1.399 (1.051–1.863)	0.021
Sagittal balance correction distance(cm)	2.634 (1.342–3.047)	0.011

QCT, quantitative computed tomography; VBQ, MRI-based vertebral bone quality; SFTT, subcutaneous fat tissue thickness; OR, odds ratio; CI, confidence interval. Statistical signifcance was set at *P* < 0.05.

### 3.4 Model robustness validation

Internal validation was performed to assess the risk of model overfitting. The five-factor logistic regression model constructed based on data from all 134 patients yielded an original AUC of 0.978. Ten-fold cross-validation yielded a mean AUC of 0.949 for the model. In addition, the AUC of the model reached 0.941 after correction via 1,000 bootstrap resampling iterations. These findings demonstrate that the established predictive model exhibits favorable robustness; its predictive performance does not decline markedly despite the limited sample size of the dataset, indicating a low risk of overfitting.

### 3.5 ROC curve analysis

Through further research and analysis, ROC curve analysis showed that correction degree of kyphosis (AUC=0.881, *P* = 0.001), Sagittal balance correction distance (AUC=0.834, *P* = 0.001) were potential risk factors with good accuracy (Table 5). correction degree of kyphosis > 20.23°(Cut-off value), Sagittal balance correction distance >2.92 cm (Cut-off value) were indicating good clinical efficacy. The remaining parameters such as SFTT, VBQ, and QCT have all obtained Cut-off values, which have certain significance in guiding the clinical prognosis of degenerative scoliosis.

**Table 5:** Sensitivity, specificity, AUC, and cut-off of predictors.

Variable	Sensitivity	Specificity	AUC	Cut-off	*P*-value
Correction degree of kyphosis (°)	91.2	80.7	0.881	20.23	0.001
Sagittal balance correction distance(cm)	78.7	80.6	0.834	2.92	0.001
SFTT(mm)	96.1	58.3	0.740	13.5	0.001
VBQ	53.7	93.5	0.669	2.88	0.001
QCT(mg/cm^3^)	83.5	96.7	0.958	104.6	0.001

QCT, quantitative computed tomography; VBQ, MRI-based vertebral bone quality; SFTT, subcutaneous fat tissue thickness; AUC, area under the curve. Statistical signifcance was set at *P* < 0.05.

## 4 Discussion

This study identified five independent predictors of clinical prognosis in patients undergoing posterior decompression and fusion for degenerative lumbar scoliosis. Multivariate analysis demonstrated that a greater degree of kyphosis correction, a larger sagittal balance correction distance, a higher QCT value, a lower VBQ score, and a lower SFTT were significantly associated with a better functional outcome, as defined by an ODI improvement rate of ≥25%. Notably, ROC curve analysis established specific thresholds for optimal surgical efficacy: kyphosis correction >20.23°, sagittal balance correction distance >2.92 cm, QCT >104.6 mg/cm^3^, VBQ <2.88, and SFTT <13.5 mm.

In contrast to adolescent idiopathic scoliosis, surgery for degenerative scoliosis depends more on the patient's symptoms rather than the angle of scoliosis. The scope of decompression typically focuses on the compressed segment, but there is no standardized approach regarding whether to perform long or short segment fusion after decompression. Some studies suggest that short-segment fusion is feasible in patients with a stable spine, scoliosis angle <20°, and lateral slip <2 mm (22). However, in this study, all operated patients had scoliosis angles >30°and lateral slip >3 mm, leading to internal fixation beyond the parietal region of the scoliosis in addition to decompression graft fusion at the compressed segment. While fixation near the parietal region can increase local stress and potentially lead to junctional scoliosis and kyphosis progression, this procedure effectively corrects the kyphosis in patients (23, 24). Smoking is well-documented as a risk factor for poor postoperative function and residual back pain after degenerative scoliosis surgery (25). Nonetheless, smoking status showed no intergroup difference in our univariate analysis (*P* = 0.139) and was excluded from the multivariate model. This inconsistency with prior data likely stems from our cohort's female predominance with low female smoking rates, alongside limited sample size reducing statistical power.

In recent years, the application of CT scanning raw data for QCT analysis has been proposed, which provides a more accurate evaluation of bone without additional examinations (10). In the past, dual energy x-ray (DXA) was a commonly used method for detecting bone density. Due to the limitations of DXA examination, lumbar spondylosis, small joint sclerosis, and abdominal aortic calcification may all lead to false increase in BMD (10). However, QCT uses CT data to convert CT values into lumbar BMD through software and calibration models, which is more accurate (10, 26). In this study, it was also found that there is a certain correlation between QCT data and postoperative efficacy, which is a new discovery in QCT guidance for clinical prognosis.

Interestingly, there is a certain correlation between low bone density and poor postoperative outcomes. Recently, MRI based assessments of the vertebral bone quality (VBQ) were introduced (20, 27). There is a high correlation between the elevation of VBQ and interbody fusion cage sink after interbody fusion surgery. This study first discovered the correlation between vertebral bone quality and postoperative lumbar degenerative scoliosis, and the mechanism of its impact may be related to the higher fat infiltration content of the vertebral body which may affect early intervertebral fusion (27).

The Goutallier classification is a reliable efficient tool for assessing fatty infiltration of paraspinal muscles in patients (14). Patients with poor efficacy have higher fat content in the lumbar paravertebral muscles (14). Previous studies have shown that a high percentage of fat in the multifidus muscle is associated with an increased risk of high intensity pain/disability (28). Therefore, it is crucial to encourage postoperative patients to undergo early rehabilitation exercises while enhancing the strength of the lower back muscles. Recent studies have shown that SFTT at the lumbar spine level, especially at the L1-L2 level, is more effective in predicting severe changes in lower back pain than BMI (16). SFTT can distinguish between muscle mass and fat mass, which may have an impact on the efficacy of spinal surgery. We collected data in this area for the first time and found that an increase in SFTT values can reduce the efficacy of scoliosis correction surgery.

Simmons et al. (29) insisted that surgery should focus on restoring spinal sequence balance, with particular emphasis on reconstructing the lumbar physiological anterior convexity or kyphosis in patients with loss of that curvature. They noted that this is more important than correcting lateral deformity. In this study, by analyzing preoperative and postoperative clinical data, we confirmed that parameters related to correction of sagittal parameters including kyphosis and SVA were important prognostic indicators after surgery. A correction degree of kyphosis <20.23°and sagittal balance correction distance <2.92 cm indicated a poor postoperative prognosis. The above findings have important implications for the surgical treatment of degenerative scoliosis.

This study has several limitations, including a relatively small sample size—particularly in the poor prognosis subgroup (*n* = 31), which may introduce a risk of overfitting in the multivariate predictive model. However, to address this, we rigorously applied 10-fold cross-validation and bootstrap resampling, which yielded a cross-validated AUC of 0.949 and a bootstrap-corrected AUC of 0.941, demonstrating robust predictive performance despite the sample size constraint. Additionally, as a single-center retrospective analysis, the study may be subject to selection bias, and the exclusion of patients who underwent secondary revision surgery to ensure preoperative baseline consistency may limit generalizability. Finally, the follow-up duration was relatively short, underscoring the need for longer-term evaluation to confirm the surgery's sustained efficacy and the model's prognostic validity. Future prospective, multicenter studies with extended follow-up are necessary for external validation.

In conclusion, surgical treatment of degenerative lumbar scoliosis significantly alleviates pain, and long-segment fixed fusion provides better correction of the deformed spine and slows down the progression of scoliosis compared to short-segment fusion. Among the many factors that may affect prognosis, the correction of kyphosis and sagittal balance is particularly crucial for the postoperative quality of life. Compared to traditional bone density DXA measurements, QCT can better predict the postoperative efficacy of patients with degenerative scoliosis, and the discovery of VBQ and SFTT on MRI can also effectively guide the prognosis of patients with degenerative scoliosis after surgery.

## Data Availability

The datasets presented in this article are not readily available because the dataset is for non-commercial use only and requires attribution to the original author. Requests to access the datasets should be directed to tongtongf430@126.com.
